# Damage Detection for Conveyor Belt Surface Based on Conditional Cycle Generative Adversarial Network

**DOI:** 10.3390/s22093485

**Published:** 2022-05-03

**Authors:** Xiaoqiang Guo, Xinhua Liu, Grzegorz Królczyk, Maciej Sulowicz, Adam Glowacz, Paolo Gardoni, Zhixiong Li

**Affiliations:** 1 School of Mechatronic Engineering, China University of Mining & Technology, Xuzhou 211006, China; godric_guo@cumt.edu.cn; 2 Faculty of Mechanical Engineering, Opole University of Technology, 45-758 Opole, Poland; g.krolczyk@po.opole.pl (G.K.); zhixiong.li@yonsei.ac.kr (Z.L.); 3 Department of Electrical Engineering, Cracow University of Technology, 31-155 Cracow, Poland; maciej.sulowicz@pk.edu.pl (M.S.); adglow@agh.edu.pl (A.G.); 4 Department of Civil and Environmental Engineering, University of Illinois at Urbana-Champaign, Champaign, IL 61820, USA; gardoni@illinois.edu; 5 Yonsei Frontier Lab, Yonsei University, Seoul 03722, Korea

**Keywords:** damage detection, conditional CycleGAN, incremental image fusion, transfer learning

## Abstract

The belt conveyor is an essential piece of equipment in coal mining for coal transportation, and its stable operation is key to efficient production. Belt surface of the conveyor is vulnerable to foreign bodies which can be extremely destructive. In the past decades, much research and numerous approaches to inspect belt status have been proposed, and machine learning-based non-destructive testing (NDT) methods are becoming more and more popular. Deep learning (DL), as a branch of machine learning (ML), has been widely applied in data mining, natural language processing, pattern recognition, image processing, etc. Generative adversarial networks (GAN) are one of the deep learning methods based on generative models and have been proved to be of great potential. In this paper, a novel multi-classification conditional CycleGAN (MCC-CycleGAN) method is proposed to generate and discriminate surface images of damages of conveyor belt. A novel architecture of improved CycleGAN is designed to enhance the classification performance using a limited capacity images dataset. Experimental results show that the proposed deep learning network can generate realistic belt surface images with defects and efficiently classify different damaged images of the conveyor belt surface.

## 1. Introduction

Although the transformation of energy structures in China has been implemented for many years and the share of coal and fossil fuels has been declining steadily, there is still no substitute for coal in China’s industrial production. As the most important coal transport equipment, it is critical to inspect belt conveyor running status and maintain its normal operation.

The normal conveyor belt consists of compounded rubber and steel cords [1], which are used to enhance wear-resisting performance and tensile strength, respectively. However, the coal transported by belt conveyors inevitably mix with kinds of foreign bodies, such as sharp metal bars and plates and large rocks, which can damage the belt surface and even lead to major production accidents. To prevent any kind of tragedy, much research has been carried out and various approaches to damage inspection have been proposed.

Early studies focused on sensor-based damage detection methods [2], which have several limitations and are no long studied. With the development of high-performance chips and processors, researchers proposed defect detection methods based on invisible light, such as X-ray or hyperspectral. Although these methods have the advantages of high accuracy and efficiency, their disadvantages, such as being harmful to human health and high cost, are unacceptable under some circumstances, which limit their applicability. Last decade, methods based on machine vision and deep neural networks (DNN) were paid more and more attention by scholars and engineers. Machine vision-based surface detection methods for mining conveyor belt surface acquire images or image-like data by an acquisition module, then process data samples by machine learning algorithms, such as image segmentation, edge detection, histogram analysis, and Fourier transform. Delicate designed machine learning detection and classification methods based on machine vision can solve specific problems, such as belt longitudinal tear, but are vulnerable to illumination, dust, and temperature. Deep neural networks are another kind of method, which focus on architecture designing. In addition, the deep neural network can update parameters by back propagation algorithms with the training dataset of belt defect images and obtain strong ability of defect detection. Machine learning methods based on the characteristics of different datasets can be divided into three categories: (1) supervised learning (SL); (2) unsupervised learning (UL); (3) semi-supervised learning (SSL). The above image-based belt defect detection approaches depend on manually annotated datasets containing enormous damaged and non-damaged image datasets, which are defined as supervised learning. However, it has been demonstrated that supervised learning methods are extremely effective and of outstanding performance, annotated datasets are high-cost and even unavailable in some research fields despite their potential problems, such as data imbalance. On the contrary, unsupervised learning methods try to classify different classes by training the model with non-annotated datasets. Due to lack of label information, unsupervised learning methods sometimes lead to undesirable results. Sometimes semi-supervised learning methods are a compromise between SL and UL methods, which are proposed under the condition of merging their advantages.

One of the most promising generative adversarial networks for domain adaptation is the CycleGAN [3], which consists of two GANs converting images from two different domains A and B. One generator can transfer images from domain A to new samples which have similar styles in the domain B. The other generator can do the opposite. Because of the two transformations of A-to-B and B-to-A, this model is named CycleGAN. The CycleGAN transfers domain styles using unpaired images from two domains, which is more flexible and can solve the problem of paired-dataset preparation for other domain adaptation GAN models.

In this paper, we propose a novel supervised deep neural network based on conditional CycleGAN to detect belt defects and address data imbalance problem. The main contributions in this paper are described as follows:

(1) Conditional and multi-classification: the multiple classifier and embedded labels [4] are established and merged into the original CycleGAN model, so that the proposed network has the ability of belt damage classification and controlled class generation;

(2) Incremental image fusion ratio: the merged image, which would be used as a training discriminator, is fused by a gradually varied ratio of real image to fake image. Since the discriminator would be trained by fresh data in each training step, the classification network has stronger generalization ability and would not tend to be over-fitting;

(3) Hinge loss and transfer learning (TL): to accelerate the training process and make it more stable, the hinge loss and feature based transfer learning are applied in the network.

The rest paper is organized as follows. Section 2 introduce related works of generative adversarial network and the background of bel defect detection methods based on machine vison and deep learning. The proposed multi-classification conditional CycleGAN algorithm is demonstrated in Section 3, and experiments and corresponding results are presented in Section 4. Finally, Section 5 concludes this paper and discusses future works.

## 2. Related Work

Recent literatures relevant to this paper can be classified into two research streams: belt surface damage detection methods and generative adversarial networks.

### 2.1. Belt Surface Damage Detection Methods

The conveyor belt with heavy loads used in power plants, the mining industry, and chemical field consists of several layers and steel cords which usually are processed with a variety of chemical components. The goal of conveyor belt damage detection methods is to inspect the surface or inside of the conveyor belt and classify these damages into several categories, such as crack or tear. A comprehensive review about conveyor belt damage detection has been studied in [5], which researched most of the popular conveyor belt damage detection methods and discussed the advantages and disadvantages between these methods. The damaged belt detection methods can be classified into magnetic [6,7], X-ray [8,9], and spectrum [10] methods. Except for above common belt damage detection methods, weak damage detection methods based on stochastic resonance [11,12,13] have the potential to be applied in coal mining fields.

(1) Traditional vision-based belt damage detection methods. With the development of high-resolution cameras and machine learning algorithms, these methods make online real-time inspection and classification possible. Various well-designed defect detection methods for conveyor belt surface are proposed. Traditional machine vision methods focus on processing images captured by industrial CCD/CMOS cameras and applying classical machine learning algorithms. Li and Miao [14] proposed a novel longitudinal tear detection method based on Single Scale Retinex (SSR) algorithm. The enhanced image contrast makes tear region segmentation efficient and accurate. To alleviate the uneven lighting condition and enhance image contrast in the underground, a novel AdaBoost algorithm is proposed in [15] based on multiple weak classifiers with Haar feature. The experimental result shows that the AdaBoost algorithm is effective in dramatic lighting changes. To address the conveyor belt damage classification problem, Hao and Liang [16] proposed an improved multi-class support vector machine (SVM) based on features obtained from preprocessed images. Visual attention mechanism is introduced to extract underlying salient features and multi-class SVM is trained with improved kernel function. Despite the possibility of misclassification, the accuracy is relatively high. Since they are obtained in a dusty and complex environment, the images captured by industrial cameras with normal lighting are undesirable for model training and damage detection.

(2) Lasers and hyperspectral camera assisted belt damage detection methods. To improve the image quality, scholars proposed numerous image fusion algorithms with auxiliary equipment, such as linear lasers and hyperspectral cameras. Li et al. [17] adopted industrial camera combined with multiple sets of lasers to capture belt surface images and proposed a novel belt tear detection method. Then, the segmentation process based on local adaptive threshold and improved Sobel operator is applied to detect tear points. Lv et al. [18] proposed an improved gray-gravity center method based on laser line images. The advantage of assisted laser methods converts blurry belt tear features into several fracture points in parallel laser lines, which can alleviate the influence of environment condition and make tear detection effective. In [19], a fusion method of Integrative Binocular Vision Detection (IBVD) based on infrared and visible image is proposed to detect tear region in conveyor belt. A well-designed light path is split into visible CCD camera and infrared CCD camera. Visible and infrared images are processed and fused simultaneously, which makes use of both cameras’ advantages. Yu et al. [20] analyzed IBVD and proposed Dual Band Infrared Detection (DBID) method based on mid- and long-infrared cameras. The damage features are obtained from mid-infrared images and tear features are suggested in long-infrared image. The experimental result shows that tear region and tearing precursor can both be detected effectively. An improved multi-spectral analysis method with a fast Fourier transform (FFT) algorithm was proposed in [10], which can establish high-contrast thermal image and locate tear regions. The above methods apply traditional machine vision algorithms with or without auxiliary equipment, which can make tear detection possible and effective to a certain extent, but it contains limitations.

(3) Deep learning-based belt damage detection methods. Last decade, deep neural networks (DNN) with the advantages of strong feature extraction ability and flexible network architectures have attracted more and more attention. Some researchers apply deep learning algorithms to belt tear detection. Liu et al. [21] established a belt image dataset and proposed a belt edge detection method based on a Holistically-Nested Edge (HED) network, which can inspect belt deviation with high precision and be applied to production scenarios. An improved YOLOv3 replace the original backbone, Darknet53, as EfficientNet was proposed in [22] to detect and locate conveyor belt damage region in real-time. Due to the balanced network architecture in depth and image resolution, it has reached a good balance between detection speed and mAP (mean Average Precision). Qu et al. [23] built a deep convolution network to extract belt surface features in different image scales, which classified real-time frame into certain conveyor belt damage. The proposed model outputs three scale feature maps and has the ability of detecting different scale tear regions. Data augmentation was applied in [24] to establish special image dataset for conveyor belt surface. An improved weighted loss function was used to optimize the model training process.

### 2.2. Generative Adversarial Networks

The supervised learning methods are extremely dependent on labeled datasets to acquire good performance. However, there are few public large-size and high-quality labeled datasets in special domain, such as mining industry, which leads to a lack of feature extraction ability and over-fitting for training deep learning networks. Since 2014, Goodfellow et al. [25] proposed generative adversarial network (GAN), which brings up a brand new research field for generative models and provides a novel solution to address training problem of insufficient datasets. Based on the theory of generative models, lots of derivative models [26] are proposed and applied to image super-resolution and translation, face synthesis and natural language processing, etc. To generate higher image resolution, Ledig et al. [27] proposed a novel Super-Resolution GAN (SRGAN). Based on the proposed perceptual loss function, SRGAN contains the ability of image four times magnification. An image-to-image translation methods, named pix2pix, based on GAN was proposed by Isola et al. [28]. The pix2pix approach can synthesize images among various tasks and have excellent performance. The self-attention mechanism was applied in GAN and a novel SAGAN model was proposed by Zhang et al. [29]. Based on self-attention mechanism, the SAGAN can establish internal connection among adjacent regions and the quality of synthesized images was improved. Yu et al. [30] proposed a novel SeqGAN to address the problem of the poor performance of the original GAN for generating sequential data. For damaged belt surface detection, an improved GAN model was proposed in [31] based on deep convolutional GAN (DCGAN) with labels embedded in latent layer and multi-class Softmax as activation function. In addition, skip connection is used in both generator and discriminator networks, which can alleviate the vanishing gradient problem and improve training speed. GAN models have been widely applied in various domains but mining industrial and provide a novel solution to DNN train problems, such as class imbalance and insufficient dataset.

### 2.3. Discussion

However, although many approaches to detect damaged belts have been developed in the above literature, they have some common disadvantages, summarized as follows. Firstly, the X-ray/spectrum-based approaches consist of complex hardware, but the performance is poor. Secondly, the deep learning methods need abundant training samples which are barely acquirable and expensive. Finally, few research studies have focused on generative adversarial networks to synthesize artificial samples.

In this paper, a novel multi-classification conditional CycleGAN is proposed to generate realistic training images and obtain a well-trained classifier based on limited training samples. During the training procedure, to address the problem of insufficient training samples, the incremental image fusion mechanism is proposed. Then the merged images are fed into the classifier network. Based on above innovations, the proposed algorithm obtains excellent performance in capacity limited datasets.

## 3. Materials and Methods

### 3.1. Basic Theory of Generative Adversarial Networks

Generative adversarial networks, based on generative models and game theory and as a subset of deep neural networks, contain two independent networks instead of one, i.e., generator and discriminator, which is the major difference with convolutional neural networks (CNN). The generator tries to learn feature distribution in real dataset and mapping from known distribution to training dataset. The desired result for generator is producing realistic samples which are indistinguishable by discriminator. Nevertheless, the discriminator is responsible for validating if an input sample is real, or fake. The iterative training process would end until Nash equilibrium is achieved between the generator and discriminator. In this situation, any updates for the generator or discriminator could break the balance. The value function V(G, D) can be described as follows:

(1) $${min}_{G}{max}_{D}V\left(D,G\right)=E_{y∼p_{data}}logD\left(x\right)+E_{g∼p_{g}}log\left\{1-D\left[G\left(g\right)\right]\right\}$$

where, G(x) and D(x) represents the generator and discriminator, respectively. $p_{data}$ and $p_{G}$ represent training samples’ and generated samples’ distribution, respectively. The adversarial concept is reflected in the minmax optimization process. The generator tries to map simple distribution, e.g., normal distribution, to generated distribution $P_{g}$, which has the minimum divergence between distributions $P_{g}$ and $P_{data}$. In addition, the discriminator tries to maximize data sampled from real dataset and minimize data sampled from $P_{g}$, which can be treated as a binary classifier. The training process is to minimize cross entropy between distributions of $P_{g}$ and $P_{data}$. In practice, the optimizing process is iteratively performed. In addition, in each iteration, the number of optimizations for generator and discriminator are not equal, usually multiple times optimizations for discriminator and one for generator, in order to stabilize training process. In Formulation (1), objective function is indirectly represented by the expression of the discriminator, hence, GAN is a kind of machine learning methods, which belongs to implicit objective function. In fact, optimizing the objective function amounts to finding the minimal value of the Jensen–Shannon divergence between $P_{g}$ and $P_{data}$. However, if the low dimensional manifolds of these two distributions have no intersection, the Formulation (1) would always be a constant, i.e., log2, which leads to unstable training and mode collapse.

### 3.2. The Framework of the Multi-Classification Conditional CycleGAN

**Figure 1:**
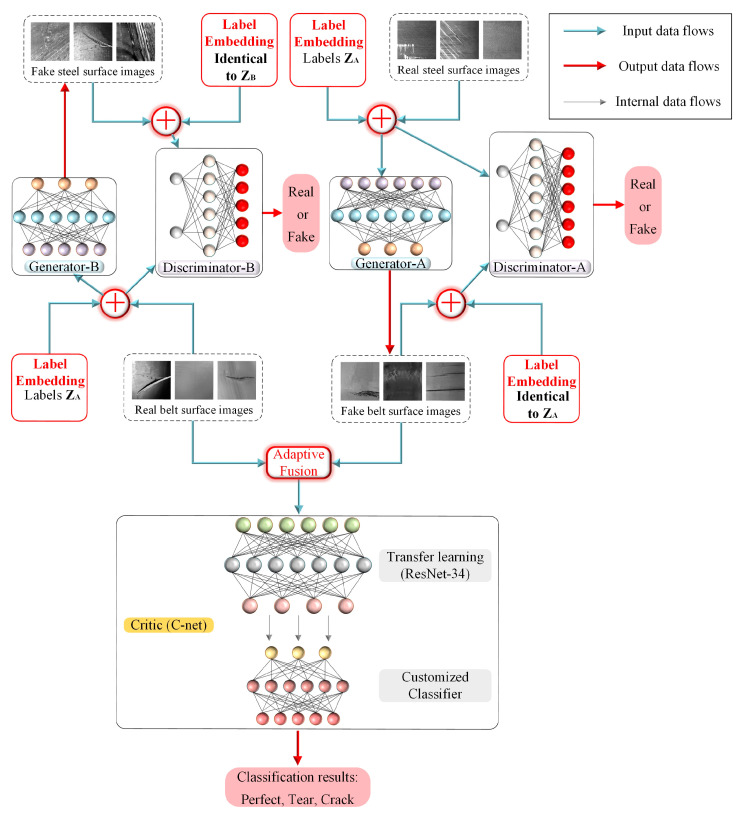
The topology of the proposed MCC-CycleGAN. The G-net transfers real steel surface images into fake belt surface images, which are fused with real belt surface images. The merged images, as new training samples, are fed into C-net.

Samples of damaged conveyor belt images are scarce and time-consuming since the acquisition environment in the mine is harsh. Inspired by CycleGAN [32], an improved Multi-Classification Conditional CycleGAN is proposed to generate damaged conveyor belt sample images and classify the belt damage. Since the damage styles between belt and steel plate are similar, we gathered the steel defect dataset from “Severstal: Steel Defect Detection” at Kaggle.com (accessed on 10 April 2022) and assume that some latent connections existed between the steel defect dataset and the conveyor belt dataset, since the damage forms between these datasets are similar except the stylistic difference. In order to address the problem of different damaged images classification, a multi-class classifier is introduced in the proposed MCC-CycleGAN, the topology of MCC-CycleGAN is shown in Figure 1.

### 3.3. The Detailed Improvements of the Proposed MCC-CycleGAN

#### 3.3.1. The Network Architectures of the MCC-CycleGAN

**Figure 2:**
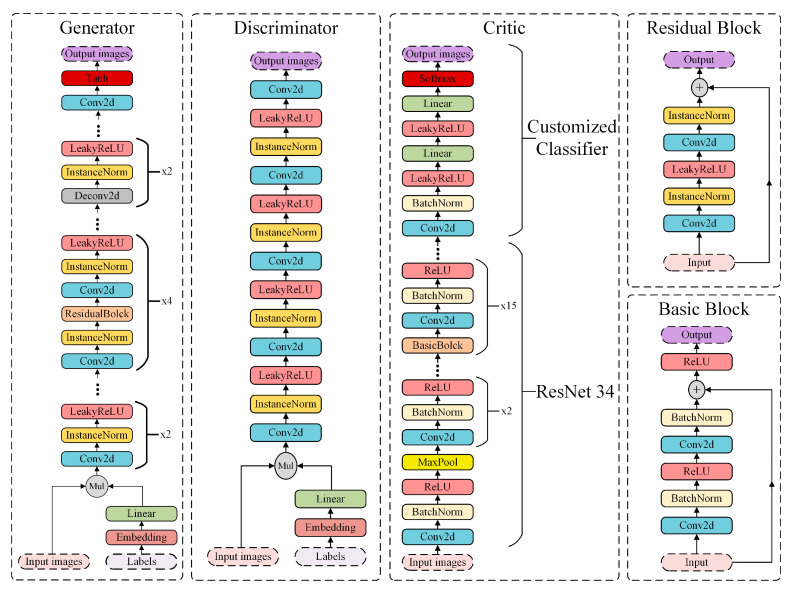
The network architecture of the Generator, Discriminator, and Critic. The residual block is used in the Generator networks, and the basic block appears in ResNet-34.

The MCC-CycleGAN consists of two Generators, two Discriminators, and one Critic. The two Generators have same network architecture but do not share weights, since one Generator is responsible for transferring damaged belt surface images into steel surface images and the other is does the opposite. The case is the same for the Discriminators. The Critic neural network is used to classify different type of damaged belt surface images, which is the essential part in this paper. The adversarial training process between Generators and Discriminators makes converted damaged steel surface images very similar to damaged belt surface images, and vice versa. The real images in the belt dataset merged with the converted steel surface images can increase the capacity of training dataset of Critic neural network and avoid over-fitting caused by insufficient training samples. The network architecture of the improved MCC-CycleGAN is as shown in Figure 2.

To make sure that the Generator can convert the desired type of damaged steel surface images, the input images combined with embedded labels are fed into the Generator network. In addition, the training samples for the Discriminator also need to be merged with embedded labels, so that the Discriminator can distinguish whether the input images are real or fake, and with correct labels or not. The shape of input for the Generator and Discriminator is identical, as (batch, channels, height, width) i.e., (batch, 1, 256, 256). The output of the Generator has same shape, since these are converted images. However, the Discriminator outputs the shape as (batch, 1), i.e., real or fake. The real images of damaged belt surface and converted images of damaged steel surface are merged by an incremental ratio which is described in Section 3.3.3. As training process goes on, the ratio of real images to converted images decreases. The Critic network can be fed with fresh-new samples, hence over-fitting can be avoided in training process with limited samples.

The ResNet-34 is adopted as backbone in the Critic, and the original classifier is replaced with special designed one, which outputs the classification results. The customized classifier has two sequential connected Linear layers, which are followed with a convolutional layer to adjust output depth of backbone network. The LeakyReLU activation layer is applied in the improved MCC-CycleGAN to avoid dying ReLU and vanishing gradient problems. The description of each network in the MCC-CycleGAN is described in Table 1. The size of input samples if grayscale image with height of 256 and width of 256. The number of classes is 3, i.e., two damaged type, tear and crack, one un-damaged type, perfect, which is encoded as one-hot code.

The proposed MCC-GAN consists of the transforming network, i.e., the Generator-A and Generator-B, Discriminator-A and Discriminator-B, and Critic, which is responsible for damage classification. Generator-A and B, Discriminator-A and B, and Critic are abbreviated as $G_{A}$, $G_{B}$, $D_{A}$, $D_{B}$, and *C*-net, respectively. The steel surface defect images dataset, as dataset A, and the conveyor belt damage images dataset, as dataset B, are established. Since the damage types in two datasets are similar but different in style, we assume that certain underlying relationship exists between the two datasets and the unpaired image-to-image transform is possible. The $G_{A}$ transforms images x with embedded labels $l_{x}$ in dataset A into $\hat{y}$ with same labels $l_{x}$ in dataset B, $G_{A}\left(x\right)=\hat{y}$, and the $G_{B}$ transforms reversely, $G_{B}\left(y\right)=\hat{x}$, where x and y are sampled from dataset A and B, respectively. In addition, $D_{A}$ and $D_{B}$ are responsible for distinguishing whether the input images are sampled from dataset A or B, or converted by $G_{A}$ and $G_{B}$. The objective is to make $x\approx \hat{x}$ and $y\approx \hat{y}$ as close as possible, so that $D_{A}$ and $D_{B}$ cannot tell the difference. In order to detect different type of belt surface damages, the classification network, i.e., *C*-net, is proposed to classify the real and generated samples in the training process. The generated samples transformed by $G_{A}$ can significantly increase training dataset and enhance the generalization ability of *C*-net, which learns new features in each training step.

#### 3.3.2. The Improved MCC-CycleGAN Loss Function

The loss function improved MCC-CycleGAN consists of the conditional CycleGAN loss, ${ℒ}_{GAN}$, and multi-class classification loss, ${ℒ}_{MC}$. The adversarial objective function with hinge loss can be described as:

(2) $${ℒ}_{GAN}\left(G_{A},D_{B},X,Y\right)=-E\left\{D_{B}\left[G_{A}\left(x,l_{x}\right),l_{x}\right]\right\}+E\left\{max\left[0,1-D_{B}\left(y,l_{y}\right)\right]\right\}+E\left\{max\left[0,1+D_{B}\left(G_{A}\left(x,l_{x}\right),l_{x}\right)\right]\right\}$$

where, x∈X is sampled image from domain X and $l_{x}$ is the embedding vector corresponding to label of x. $G_{A}$ tries to transfer image x with label $l_{x}$ to $\hat{y}$, which looks similar to image y in domain Y. In addition, $G_{B}$ is responsible for distinguishing whether the input image $G_{A}\left(x,l_{x}\right)$ with label $l_{x}$ is the fake image transferred by $G_{A}$, or the input image y with label $l_{y}$ is the real image sampled from domain Y. The hinge loss only punishes positive samples which less than 1 and negative samples which greater than −1, and the formulation is much easier than original loss. Hence the training process is much faster and more stable. In addition, the ${ℒ}_{GAN}\left(G_{B},D_{A},X,Y\right)$ is similar as above.

The cycle consistency loss function can be described as:

(3) $${ℒ}_{cyc}\left(G_{A},G_{B}\right)=E_{x\in X}\left\{∥G_{B}\left[G_{A}\left(x,l_{x}\right),l_{x}\right]-x∥\right\}+E_{y\in Y}\left\{∥G_{A}\left[G_{B}\left(y,l_{y}\right),l_{y}\right]-y∥\right\}$$

where the image x sampled from domain X with label $l_{x}$ is converted to fake image $G_{A}\left(x,l_{x}\right)$, which is transferred back to $G_{B}\left[G_{A}\left(x,l_{x}\right),l_{x}\right]$. If the $G_{A}$ and $G_{B}$ are well trained, the L1-norm between image $G_{B}\left[G_{A}\left(x,l_{x}\right),l_{x}\right]$ and x should be small enough. Same to $G_{A}\left[G_{B}\left(y,l_{y}\right),l_{y}\right]$ and y, minimize ${ℒ}_{cyc}\left(G_{A},G_{B}\right)$ can ensure the style consistency between domain X and Y.

The multi-classification loss for the Critic can be described:

(4) $${ℒ}_{critic}\left(C\right)=E_{z\in Mix\left(X,\;\hat{X}\right)}\left[\log C\left(z\right)\right]$$

where $\hat{X}$ is the domain which contains images $G_{B}\left(y,l_{y}\right)$. The input image z is merged by Formulation (2), and the Critic loss is multi-class cross entropy. In the experiments of different loss choices, such as mean square error (MSE) and cross entropy, the latter has better performance.

The final loss for MCC-CycleGAN can be described as:

(5) $$ℒ\left(G_{A},G_{B},D_{A},D_{B},C\right)={ℒ}_{GAN}\left(G_{A},D_{B},X,Y\right)+{ℒ}_{GAN}\left(G_{B},D_{A},Y,X\right)\;+\lambda {ℒ}_{cyc}\left(G_{A},G_{B}\right)+{ℒ}_{critic}\left(C\right)$$

where λ is used for adjusting the punishment among generator, discriminator, and critic:

(6) $$min_{G_{A},G_{B},C}max_{D_{A},D_{B}}ℒ\left(G_{A},G_{B},D_{A},D_{B},C\right)$$

By minimizing and maximizing the above loss function, the networks of Generator, Discriminator, and Critic can obtain appropriate weights and the training process is much more stable and faster. A detailed analysis is presented in Section 4.

#### 3.3.3. The Image Fusion Strategy of the MCC-CycleGAN

**Figure 3:**
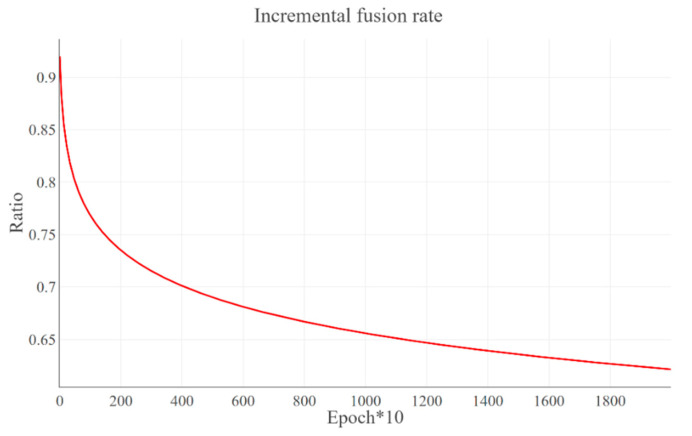
The curve of the incremental fusion rate. With the training progress, the ratio of x to $\hat{x}$ decreases.

The *C*-net is fed with fusion images which are obtained by merging real belt surface images x with transferred steel surface images $G_{B}\left(y\right)=\hat{x}$ as an incremental fusion rate, Ratio, which can be described as:

(7) $$Ratio=1-\frac{\log\left(epoch*10+k\right)}{e^{3}}$$

where k is a constant, and we set k=5.0 based on multiple experiments. The Ratio curve is as shown in Figure 3. In addition, the fusion image can be obtained by following formulation:

(8) $$img_{mix}=ratio\cdot a+\left(1-ratio\right)\cdot img\_g_{A}$$

In the initial stage of training, the *C*-net is mainly fed with real belt surface image, which makes the training of Critic network stable but slow progress. As the training goes on, the proportion of transferred steel surface image, $G_{B}\left(y\right)=\hat{x}$, increases and the *C*-net is trained with new fusion images. Due to the proposed incremental image fusion mechanism, the *C*-net prevent to be over-fitting in training and gain better generalization ability in testing.

#### 3.3.4. Feature Based Transfer Learning and Fine-Tuning

Feature based transfer learning [33] is applied to speed up the training process and improve the Critic network performance. The basic feature extraction layers of ResNet-34 [34] and their pretrained weights are used and the customized classifier is designed, which receives extracted basic features and make the classification. The pretrained ResNet-34 model contains excellent underlying feature extraction abilities, which are used for extracting simple features, such as edges, shapes, and textures. These basic features also exist in damaged belt surface images. To shorten training time and improve Critic model performance, the pretrained model of ResNet-34 is selected as feature extraction model and the followed special customized module containing multiple layers are treated as classifier.

Since the loaded ResNet-34 model is trained for CIFAR-1000, the number of layers to be frozen needs to be determined. Verified by multiple experiments, the first seven blocks of convolutional modules which contain basic features are frozen and the rest of the layers are trainable. The experimental results described in Section 4 have proved the performance and efficiency of the proposed Critic model. In general, the trainable parameters can be reduced to around half and the performance of the Critic network almost keeps the same.

### 3.4. The Training Procedure of the MCC-CycleGAN

In order to present the network training process thoroughly, the pseudocode of training is described in Algorithm 1. For each batch of the training procedure, the generator is trained 3 (n=3) times and the discriminator or the Critic is trained once, since the training for generator is much more difficult than training for other networks. In addition, the input for Critic is the mixed images, which is described in Section 3.3.3. In addition, the specific implementation of fine-tuning is discussed in Section 3.3.4.

**Algorithm 1.** MCC-CycleGAN training process. The pseudocode of the proposed network training process.1:**Input:** hyperparameters (batch size k, epochs e, times of generator training n, learning rate r), location of dataset A and B2:$\mathrm{Establish}\;\mathrm{and}\;\mathrm{initialize}\;\mathrm{models}:\;G_{A}$$,\;G_{B}$$,\;D_{A}$$,\;D_{B}$ and C, setup optimizer: Adam,

Load training dataset A


 and B


, samples a∈A and samples b∈B

3:**For** epoch=1 **to** e **do**4:**For** t=1 **to** n **do**5:**Train** $G_{A}\;\mathrm{and}$ $G_{B}:$$\;\mathrm{freeze}\;\mathrm{parameters}\;\mathrm{of}\;D_{A}\;\mathrm{and}\;$$D_{B}$, generate fake images$img\_g_{A}=G_{A}\left(b\right)$$\;\mathrm{and}\;img\_g_{B}=G_{B}\left(a\right)$$,\;\mathrm{compute}\;loss_{GA}$$,\;loss_{GB}$ based$\mathrm{on}\;\mathrm{Equation}\;(2),\;\mathrm{and}\;loss_{cycle}$ based on Equation (3), then update parameters of$\mathrm{model}\;G_{A}\;\mathrm{and}$
 
$G_{B}$
6:
**end for**
7:**Train** $D_{A}\;\mathrm{and}$ $D_{B}:$$\;\mathrm{freeze}\;\mathrm{parameters}\;\mathrm{of}\;G_{A}\;\mathrm{and}$ $G_{B}$, generate fake images$img\_g_{A}=G_{A}\left(b\right)$$\;\mathrm{and}\;img\_g_{B}=G_{B}\left(a\right)$$,\;\mathrm{compute}\;loss_{GA}$$,\;loss_{GB}$ based on$\mathrm{Equation}\;(2),\;\mathrm{then}\;\mathrm{update}\;\mathrm{parameters}\;\mathrm{of}\;\mathrm{model}\;D_{A}\;\mathrm{and}$
 
$D_{B}$
8:**Train** C:$\;\mathrm{compute}\;\mathrm{the}\;\mathrm{input}\;\mathrm{fusion}\;\mathrm{image}\;img_{mix}$ based on Equation (8), feed$img_{mix}$ into C$,\;\mathrm{compute}\;loss_{critic}$ based on Equation (4), then update parameters

of model C

9:
**end for**


## 4. Results

### 4.1. The Hardware Framework of the Conveyor Belt Damage Detection System

**Figure 4:**
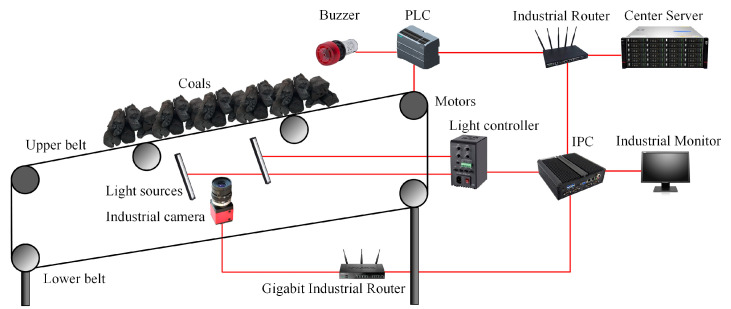
The hardware framework of the conveyor belt damage detection system.

The conveyor belt surface damage detection system consists of an image acquisition module, a transmission module, and a data processing and execution module. The hardware of each module is demonstrated in Figure 4. The image acquisition module includes the linear industrial camera, gigabit industrial router, linear light source, and controller. The images acquired by image acquisition module is transmitted to data processing module, which consists of industrial personal computer (IPC) with high-performance graphics processing unit (GPU). The postprocessing results are transmitted to execution module by transmission module, which usually consists of routers and ethernet cables. Alert or shutting down can be executed in execution module, which consists of center server, Programmable Logic Controller (PLC) and buzzers. The whole system can be shut down by PLC when the halt signal emits. If multiple cameras are deployed, gigabit industrial routers are needed to collect and transmit images from different cameras to one or more IPCs. Normal industrial cameras, e.g., 2.0 Mega Pixels industrial camera, are capable to capture clear belt images. Considering the fast speed and large width of conveyor belt, the linear industrial camera is adopted to acquire high-resolution belt surface image. So, a high-brightness linear light source is used to provide uniform and stable lighting illumination. Multiple cameras are connected to a gigabit industrial router, which serves as a data exchange center and forwards multiple image streams to (IPC). High-performance GPU is installed in IPC, which performs neural network forward propagation in real-time.

In training stage, GAN model is trained in data processing server contained several high-performance GPUs, which still takes hundreds of hours. Signals would be sent to execution module to ring the alarm or shutdown the conveyor as long as the IPC detects any conveyor belt damage. The width of conveyor belt is 1.2 m, so the linear industrial camera with wide-angle lens installed at a suitable distance can acquire high-resolution images. One set of the belt damage detection devices is deployed in the simulation environment, but multiple sets of the proposed belt damage detection devices can be deployed every few hundred meters for vulnerable regions in the production environment.

**Figure 5:**
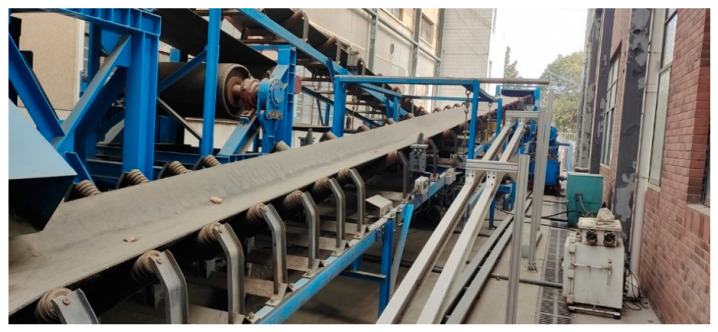
The belt conveyor in the laboratory simulation environment.

The improved MCC-CycleGAN is implemented by Pytorch and trained with NVIDIA RTX3070 8G. The steel image datasets are gathered from “Severstal: Steel Defect Detection” at Kaggle.com (accessed on 10 April 2022). Samples in the steel dataset are 1600 × 256 high-resolution labeled images, which consist of four defect types: spot, crack, scratch, and tear. The original samples in the steel dataset are segmented into 256 × 256 images. The tear defect, which is desired and corresponding to the tear in belt damage dataset, is few centimeters long and several millimeters width. The scratch defect is similar to the tear and may be multiple parallel lines, which only damages the steel surface. The crack defect involves large region on the steel surface, which may present as metal spalling. The spot defect is in millimeter-level and undesired for belt damage detection and has been removed by data cleaning, since the conveyor belt damage detection system does not need to detect micro defects on the belt surface. The defects of tear and scratch in steel dataset are intended to transfer to tear defect in conveyor belt dataset, and the crack defect in steel dataset to correspond to the crack in the belt dataset. After the preprocessing of data cleaning and one-hot encoding labeling, the custom steel dataset is prepared. The steel image dataset consists of the damaged steel surface images which are most similar to the damaged belt surface images. In addition, the belt surface images in the training dataset B are captured with industrial CMOS camera in the laboratory simulation environment, which is shown in Figure 5. The industrial camera and light sources are installed under the conveyor belt since the conveyor belt surface is covered with coals.

### 4.2. The Experimental Results and Comparisons

The dataset A and B are described in Table 2. We carefully selected an image dataset and preprocessed belt surface images. Since the belt images are captured in the laboratory, the undesirable images were excluded and annotations were added. To reduce the GPU memory usage, the color images were converted to grayscale. The datasets were split into training and testing dataset by a ratio of 4 to 1.

**Figure 6:**
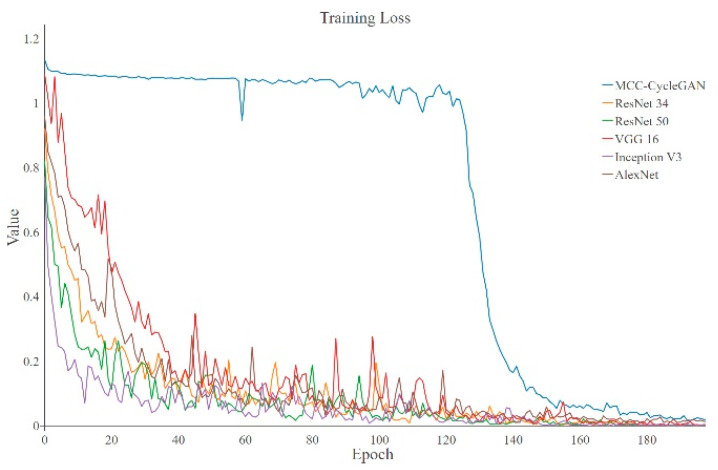
The training losses of the MCC-CycleGAN and other classical algorithms. The loss of proposed MCC-CycleGAN fluctuates around 1.1 and makes no progress, and losses of other contrastive algorithms decrease fast. At the end of training, losses of all networks stay stable and tend to be zero.

**Figure 7:**
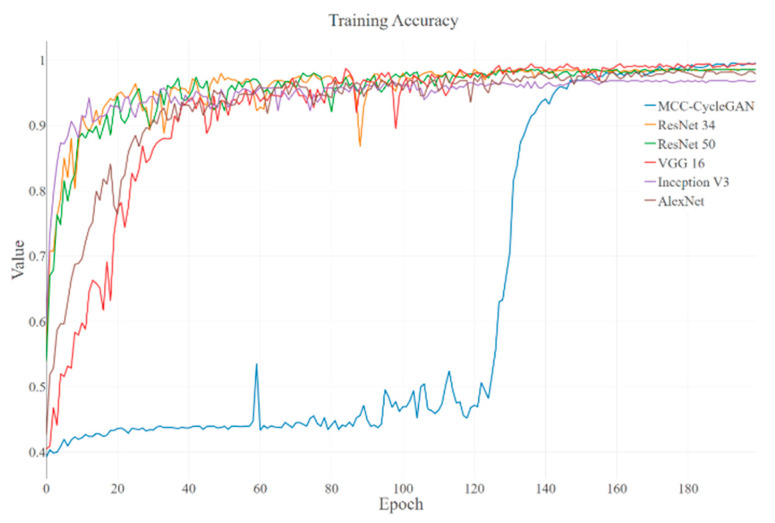
The training accuracies of the MCC-CycleGAN and other classical algorithms. The accuracies have the corresponding tendency with the losses. The accuracy of the proposed MCC-CycleGAN start to rise at epoch 130 and obtain similar accuracy compared to other contrastive networks.

The training loss and accuracy curves of the proposed MCC-CycleGAN and other comparative classical deep learning networks are shown in Figure 6 and Figure 7, respectively. We can see from Figure 6 that except of the proposed MCC-CycleGAN, other algorithms loss curves drop fast and tend to be stable at epoch 110. Combined with Figure 7, accuracies of contrastive algorithms increase rapidly because of insufficient training. The reason why the proposed MCC-CycleGAN converges slowly can be explained as follows. The proposed MCC-CycleGAN contains two sub models for sample generation, which can supplement the insufficient samples in custom dataset. In addition, considering the proposed image fusion strategy, the MCC-CycleGAN model is fed with new generated samples before 100 epochs. After 100 epochs, the MCC-CycleGAN model is trained to a certain extent and the proportion of generated image is decreasing, from 0.9 to 0.65. The training process tends to be stable after 120 epochs and the loss of the proposed MCC-CycleGAN model is drastically reduced. So, the low training loss and high training accuracy occur at 140 epochs, which is slower than other comparative networks.

**Figure 8:**
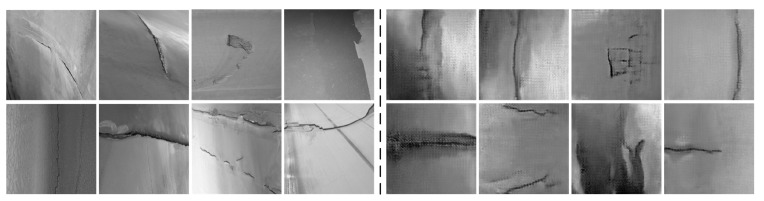
The samples from real belt surface images and transferred images from the steel surface dataset. Images on the left side are sampled from real belt dataset, and the images on the right side are transferred ones.

The training loss of proposed MCC-CycleGAN keeps relatively high and accuracy keeps relatively low than other contrastive algorithms, most likely because the low-quality generated damaged belt surface images and the incremental fusion rate, which also is the key factors of preventing over-fitting. We can inference that the proposed CycleGAN generates high-quality images around epoch 140, since the training loss and accuracy tend to be stable. Some transferred samples are shown in Figure 8. Table 3 shows the training losses and accuracies of each algorithm at the end of training procedure. Since the proposed MCC-CycleGAN contains cycle training strategy, two sub models of GAN (Generator-A and Discriminator-A, and Generator-B and Discriminator-B) and a Critic model, the training process is more time-consuming, which is revealed in Table 3. However, the cumbersome model is designed for training a better Critic model and generating new samples; the Critic model is needed and two sub models of GAN are excluded in the prediction process. So, the test FPS is quite acceptable, and the proposed Critic model can satisfy the requirement in industrial application. In addition, during the training process, the proposed MCC-CycleGAN algorithm can generate new samples, which could be used for training other algorithms.

**Figure 9:**
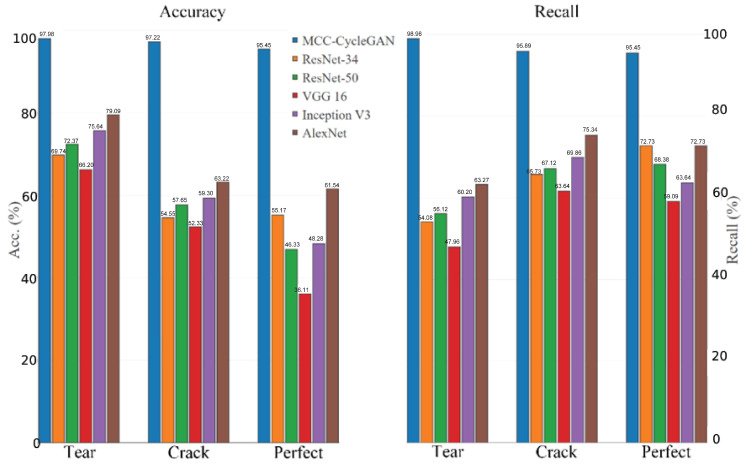
The results of the accuracies and recalls of all networks.

The results of above networks in test dataset are demonstrated in Figure 9 and Table 4. It can be seen that mean average precision (mAP) of the proposed MCC-CycleGAN reaches 96.88% and the proposed network performs excellent in test dataset. However, the best mAP in all contrastive networks does not pass 70%, which cannot satisfy the application requirements.

As we can see from Figure 9, Table 3 and Table 4, all algorithms obtain excellent training accuracies, but only the proposed MCC-CycleGAN performs well in test set. Hence, except for the proposed neural network, other classical networks suffer severe over-fitting. In general, the excellent performance of the proposed MCC-CycleGAN due to outstanding network architecture and the incremental image fusion mechanism.

### 4.3. Application of the Proposed MCC-GAN

**Figure 10:**
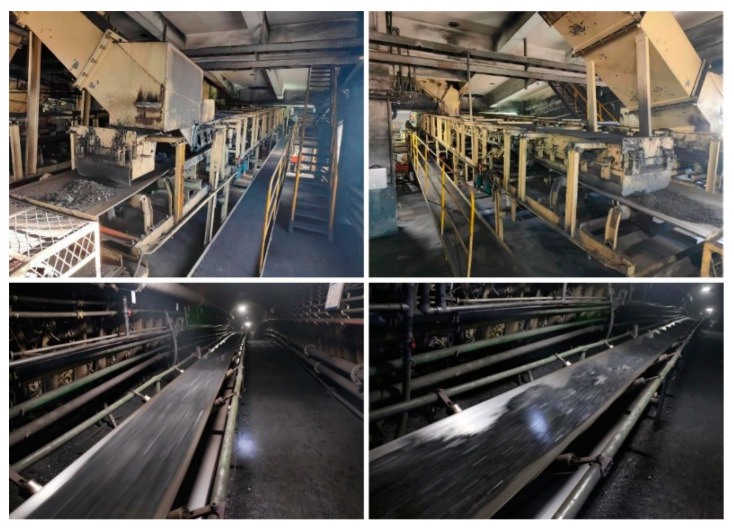
The application of the belt damage detection system.

The proposed MCC-CycleGAN detection system, shown in Figure 10, has been applied in mining industrial to inspect the state of belt conveyor surface in real-time. The proposed algorithm can detect and classify the different damage type of conveyor belt surface, and alert the workers timely when damage happens. The application of the detection system can significantly reduce work intensity of workers and detect belt damages effectively.

## 5. Conclusions

In this paper, an improved Multi-Classification Conditional CycleGAN is proposed to address the detection problem of damaged conveyor belt surface images. The proposed MCC-CycleGAN has the advantages of high-performance, fast detection speed, and the ability to generate new samples. The generated samples improve the generalization of the Critic model and can be used to the training process for other deep neural networks. By introducing embedded label vectors and extra Critic network, the proposed network can make necessary image style transferring and detect damaged belt surface images in real-time. Using the proposed incremental image fusion mechanism, the proposed MCC-CycleGAN can obtain excellent performance with very few training samples, which is almost impossible for other classical convolutional neural networks. However, the proposed MCC-CycleGAN model is cumbersome and requires long training time, which limits the model flexibility. Hence, future work can be focused on designing lightweight architecture and model compression to reduce training time-consumption.

The images from damaged belt surface are blurry and full of noise, which affects the detection effect. In the future works, we would propose a novel image enhancement algorithm based on Generative Adversarial Networks to address the problem caused by poor image quality and improve detection performance.

## Figures and Tables

**Table 1:** The description of each network in the MCC-CycleGAN.

Network	Input Size	Output Size	Trainable Parameters	Pretrained
G-A	(Batch size, 1, 256, 256)	Identical to input	9,523,319	No
G-B		Identical to input		No
D-A		(Batch size, 1)	6,721,426	No
D-B		(Batch size, 1)		No
C-net		(Batch size, 3)	15,279,936	Partial

**Table 2:** The description of the image dataset A and B.

Dataset	Image Size	Capacity	Ratio	Color Image	Annotation
A	(256, 256)	1532	4:2:4 (Tear: Crack: Scratch)	Yes	Yes
B	(256, 256)	468	5:3:2 (Tear: Crack: Perfect)	No	Annotated manually

**Table 3:** The comparison of each algorithms.

	MCC-CycleGAN	ResNet-34	ResNet-50	VGG 16	Inception v3	AlexNet
Loss	0.01367	0.00078	0.00079	0.00265	0.00098	0.01571
Acc.	99.53%	98.56%	98.56%	99.42%	96.83%	97.84%
Time consumption for training (hour)	12.6 h	4.2 h	5.4 h	4.6 h	6.1 h	7.8 h
Test FPS	44.3	47.5	41.6	31.3	37.4	40.1

**Table 4:** The test results of the proposed MCC-CycleGAN and other networks.

Evaluation	Proposed	ResNet-34	ResNet-50	VGG 16	Inception v3	AlexNet
mAP	0.969	0.598	0.590	0.515	0.611	0.681
Macro-F1	0.968	0.611	0.603	0.524	0.620	0.686

## Data Availability

Not applicable.
